# Engineering Electrospun Nanofibers for the Treatment of Oral Diseases

**DOI:** 10.3389/fchem.2021.797523

**Published:** 2021-12-20

**Authors:** Yuanfei Wang, Yingnan Liu, Xiaopei Zhang, Na Liu, Xixi Yu, Meihua Gao, Wanchun Wang, Tong Wu

**Affiliations:** ^1^ Qingdao Stomatological Hospital Affiliated to Qingdao University, Qingdao, China; ^2^ Institute of Neuroregeneration and Neurorehabilitation, Qingdao University, Qingdao, China; ^3^ Qingdao Medical College, Qingdao University, Qingdao, China; ^4^ Department of Cosmetic and Plastic Surgery, Affiliated Hospital of Qingdao University, Qingdao, China

**Keywords:** electrospun nanofibers, structures, biofunctions, oral diseases, translational application

## Abstract

With the increase of consumption of high-sugar foods, beverages, tobacco, and alcohol, the incidence rate of oral diseases has been increasing year by year. Statistics showed that the prevalence of oral diseases such as dental caries, dental pulpal disease, and periodontal disease has reached as high as 97% in 2015 in China. It is thus urgent to develop functional materials or products for the treatment of oral diseases. Electrospinning has been a widely used technology that is capable of utilizing polymer solution to generate micro/nano fibers under an appropriate high voltage condition. Owing to their excellent structures and biological performances, materials prepared by electrospinning technology have been used for a wide range of oral-related applications, such as tissue restoration, controlled drug release, anti-cancer, etc. In this regard, this article reviews the application and progress of electrospun nanofibers to various oral diseases in recent years. Firstly, engineering strategies of a variety of nanofiber structures together with their resultant functions will be introduced. Then, biological functions of electrospun nanofibers as well as their applications in the treatment of oral diseases are summarized and demonstrated. Finally, the development viewpoint of functional nanofibers is prospected, which is expected to lay the foundation and propose the direction for further clinical application.

## Introduction

Oral health is very important for our daily physiological activities. As reported from the World Health Organization, oral diseases have been the main public health problems owing to the high prevalence rate (Baiju et al., 2017). The oral health condition affects many physiological functions, including sensory function, speaking, mastication, eating, and even threatens personal life safety (Baiju et al., 2017). Thus, our quality of life would be strongly impacted once oral tissue defects accompanied with various oral diseases (Edmans et al., 2020). At present, the common diseases are mainly divided into the following categories: maxillofacial defects, periodontal diseases (gingiva inflammation, periodontal ligament, alveolar bone, and cementum loss), dental pulpal diseases, and tooth hard tissue defects (Yang et al., 2017). To date, there are still a lot of challenges and opportunities in the clinical methods and related research for the treatment of the above-mentioned oral diseases. For example, periodontal disease, as one of the most common oral diseases, has been considered as the leading cause of damage to teeth and surrounding bones and soft tissues (Amid et al., 2013). The drawback is that the pathogens leading to the periodontal disease cannot be completely eliminated by current treatment strategies (Sun et al., 2013). In addition, oral cancer, as the sixth most common malignancy worldwide, is still an enormous oral health issue. The most common therapies, such as surgery, radiotherapy, chemotherapy, and/or their synergistic treatment, have shown limited ability in preventing or delaying the recurrence of various types of oral cancers (Borges et al., 2017). In this regard, seeking effective solution is the key to solve the above problems.

**GRAPHICAL ABSTRACT:**
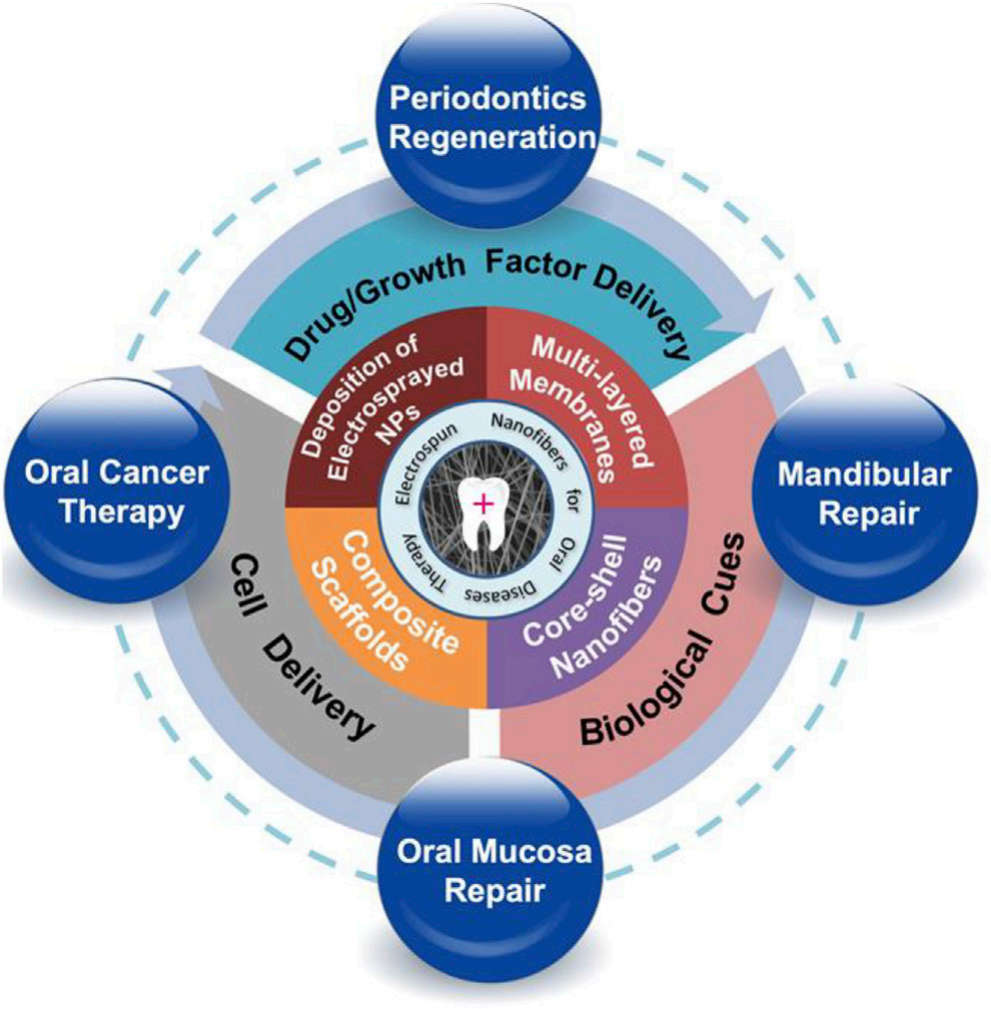


The goal of tissue engineering is to fabricate tissue/organ regeneration scaffolds with good biocompatibility, functionality, and biological activity. Cells, bioactive molecules, and scaffolds constituted the three most necessary components of tissue engineering (Langer and Vacanti, 1993; Rai et al., 2015; Borrelli et al., 2020). In recent years, with the development of tissue engineering, especially *in situ* tissue engineering (Joshi et al., 2015; Rezk et al., 2018), electrospun nanofibers have shown great potential in biomedical fields including oral disease treatment (Dong et al., 2020; Shi, 2020; Zhao and Cui, 2020). Electrospinning is a versatile manufacturing process that can produce fibrous scaffolds with a wide range of structures and biofunctions. Briefly, the polymer solution or melt is ejected and spun under a strong electric field. Then, the droplet at the tip of the needle changes from a sphere to a cone (Taylor cone), and extends from the tip to produce filaments, which makes it possible to produce nano-sized polymeric filaments (Reneker and Chun, 1996). The high porosity of electrospun nanofibers could promote the transportation of nutrients and facilitate the signal exchange between cells (Pham et al., 2006). Electrospun nanofibers are also an ideal platform for delivering bioactive molecules/drugs due to their large specific surface area (Liu et al., 2012). Besides, electrospun nanofibers can easily mimic the extracellular matrix and are capable of regulating cell behavior and morphology such as cell migration and differentiation (Hashi et al., 2007) (Schnell et al., 2007) (Xin et al., 2007). Most importantly, electrospun nanofibers have great flexibility, which can load a rich variety of components such as biologically active molecules, nanoparticles, growth factors, etc. Thus, nanofiber scaffolds with tissue repair and regeneration capabilities have attracted more and more attention and become promising alternatives to develop oral materials (Fidalgo et al., 2018).

In this review, we aim to give a comprehensive introduction of electrospun nanofibers that have been applied to the treatment of oral diseases. Firstly, we will describe the engineering strategies of electrospun nanofibers. Then, the biological functions that nanofiber scaffolds could provide, for example, the capabilities of delivering cells and/or drugs, will be introduced. Finally, we will indicate future development direction in terms of scaffold design, manufacture, engineering, and the use of electrospun nanofibers for further oral clinical application.

## Engineering Nanofibers With Specific-Controlled Structures

### Deposition of Electrosprayed Nanoparticles or Microspheres

**FIGURE 1:**
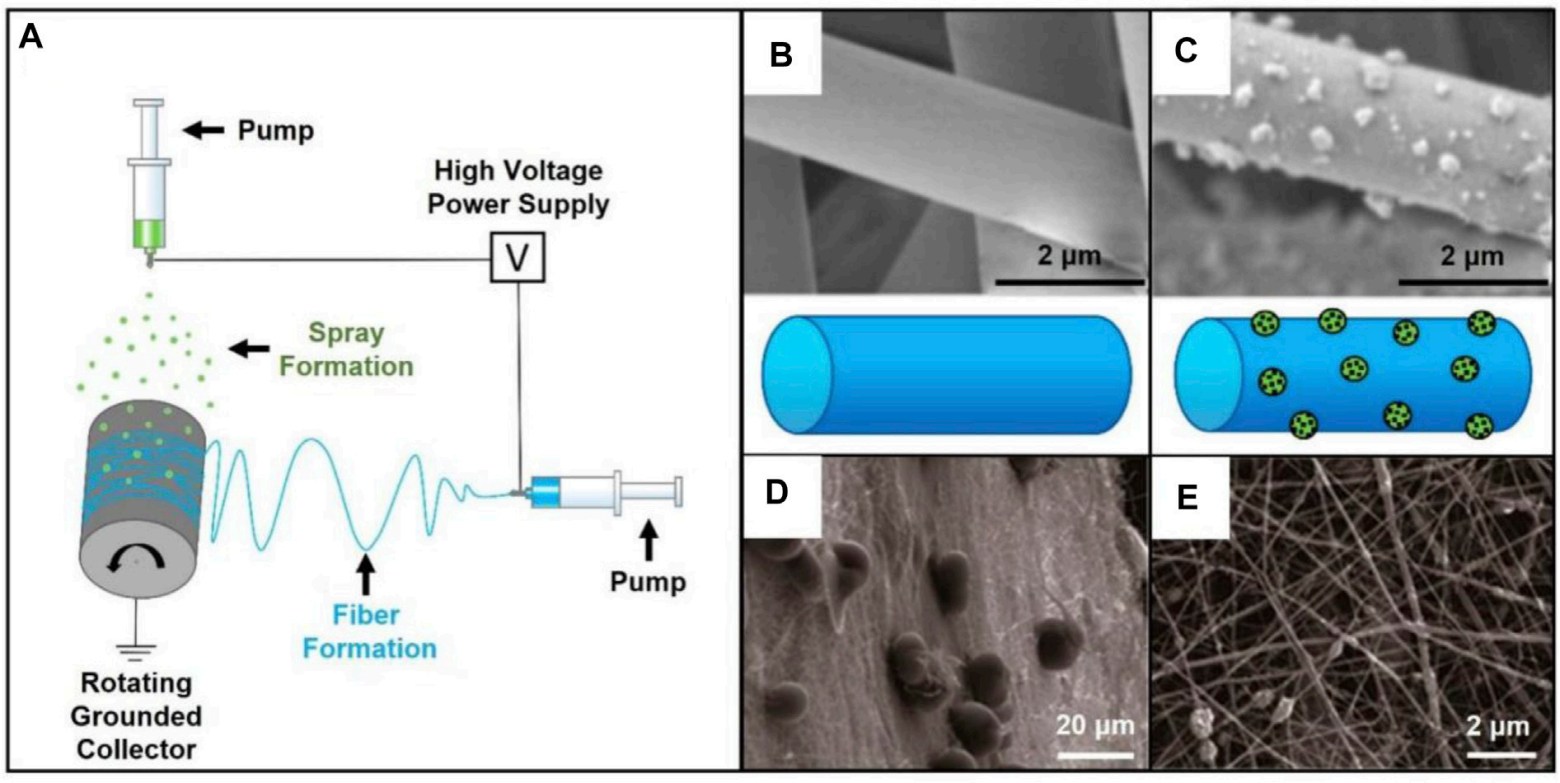
**(A)** Electrospinning setup combined with electrospray. Reproduced with permission (Higuchi et al., 2019). Copyright 2019, MDPI. **(B)** SEM image of pristine PLA/PLGA fibers. Reproduced with permission (Higuchi et al., 2019). Copyright 2019, MDPI. **(C)** SEM image of the fibers covered by n-HA particles. Reproduced with permission (Higuchi et al., 2019). Copyright 2019, MDPI. **(D)** Typical SEM image of the AMPs-loaded PLGA microspheres deposited on the barrier layer of gelatin/chitosan fibers. Reproduced with permission (He et al., 2018). Copyright 2018, MDPI. **(E)** SEM image showing the osteogenic layer made of gelatin/chitosan fibers containing n-HA. Reproduced with permission (He et al., 2018). Copyright 2018, MDPI.

Nanomaterials have been widely applied to facilitate the regeneration of damaged oral tissues (Karakoti et al., 2010; Naganuma and Traversa, 2014; Kargozar et al., 2018; Li et al., 2018; Ren et al., 2021). For example, nanoparticles have shown advantages in low price and good stability in comparison to growth factors and other bioactive molecules. Among others, nano-hydroxyapatite (n-HA) particles have been extensively used for the fabrication of guided bone regeneration (GBR) membranes due to their structural and functional properties (Lowe et al., 2020; Ren et al., 2021). As an important organic constituent part of human bone, n-HA particles provide the scaffold with excellent biocompatibility and bioactivity, thus inducing osteogenic differentiation (Jin et al., 2019). More importantly, recent studies have proved that the addition of n-HA particles can improve the mechanical property, hydrophilicity, and porosity. For instance, a biodegradable membrane used for periodontal tissue repair was fabricated *via* the electrospinning and electrospray technologies (Figure 1A) (Higuchi et al., 2019). During the electrospray process, the n-HA particles were deposited on the surface of polylactide/poly (lactic acid-co-glycolic acid) (PLA/PLGA) nanofiber membrane, thereby improving the porosity and mechanical properties of the materials (Figures 1B,C). Moreover, the PLA/PLGA membrane loaded with n-HA particles led a transformation from hydrophobicity to hydrophilicity and decrease the acidity during the degradation process of PLGA.

In another case, a GBR membrane holding antimicrobial and anti-inflammatory activities was designed and prepared through the combination of layer-by-layer electrospinning technique and electrospray (Figures 1D,E) (He et al., 2018). In this study, PLGA microspheres loaded with antimicrobial peptides (AMPs) were wrapped in the middle of two layers. In detail, the barrier layer was prepared by electrospinning a mixture of gelatin and chitosan. Another layer of gelatin/chitosan fibers were further modified with the addition of n-HA as the osteogenic layer to enhance the tensile strength. The AMPs-loaded microspheres sandwiched between the two layers endowed the scaffold with antibacterial activity. Overall, in addition to the biological functions provided by the two nanofiber layers, the deposited microspheres contributed to the increased bioactivity of the as-obtained scaffold for bone mature without bacterial infection in the process of oral bone regeneration.

### Core-Shell Nanofibrous Membrane

**FIGURE 2:**
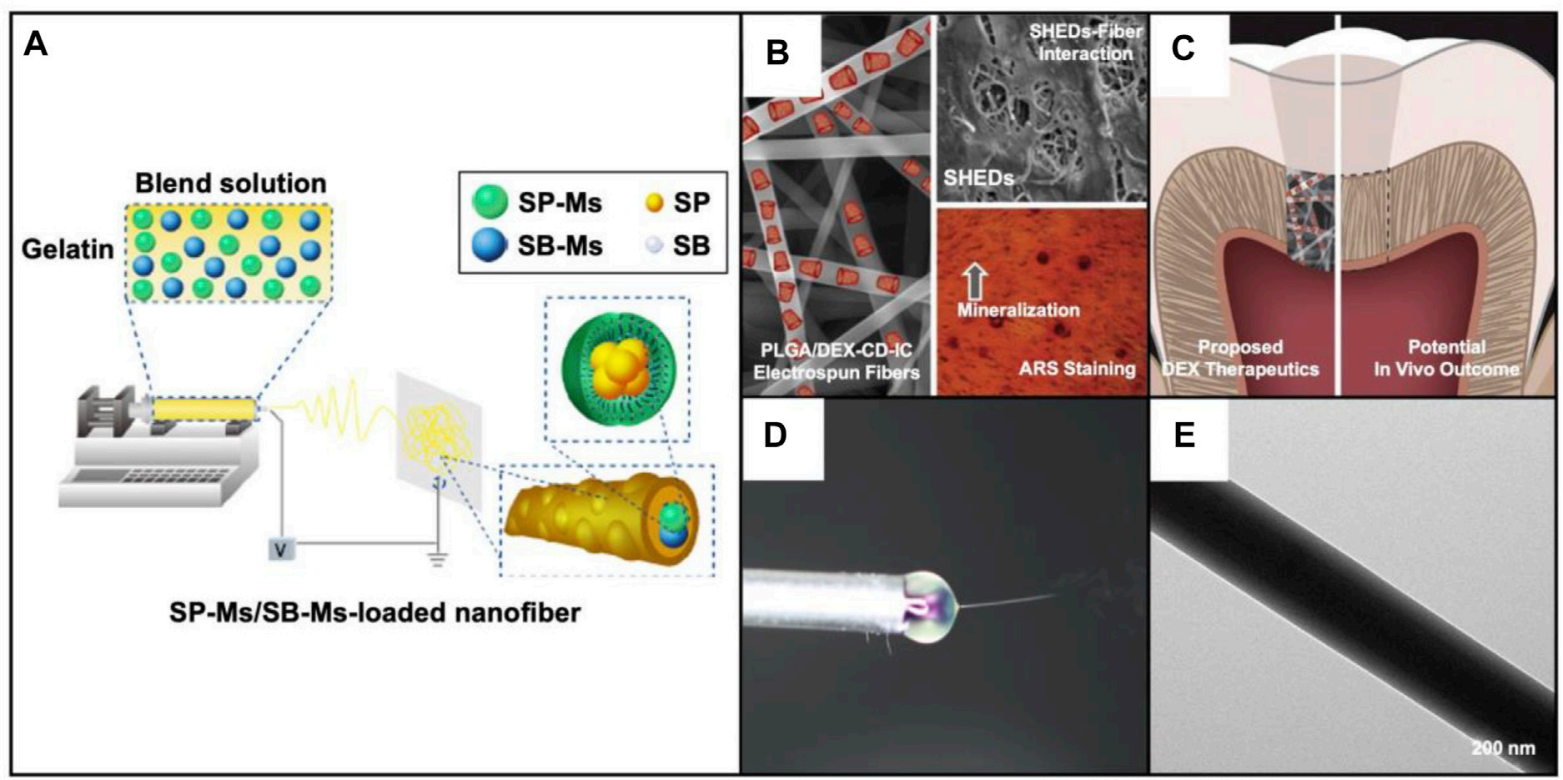
**(A)** Schematic illustration showing the fabrication of the dual-drug delivery system. SP600125 and SB203580 were separately loaded into the PEG-b-PCL micelles, and then the micelles were blended with gelatin solution for electrospinning. Reproduced with permission (Wang Y. et al., 2019). Copyright 2019, Dovepress. **(B)** SEM image together with a schematic illustration showing the PLGA fibers loaded with dexamethasone and cyclodextrin inclusion complex (PLGA/DEX-CD-IC) (left); SEM image showing the single-cell suspensions isolated from human pulp tissues seeded on the fibers (right top); Alizarin red staining showing the effect of DEX induced mineralization (right bottom). Reproduced with permission (Daghrery et al., 2020). Copyright 2020, Elsevier. **(C)** Schematic illustration showing the proposed therapeutics with the PLGA/DEX-CD-IC fibers and the potential *in vivo* outcome. Reproduced with permission (Daghrery et al., 2020). Copyright 2020, Elsevier. **(D)** Photograph of the core-shell jet at the end of spinning needle. Reproduced with permission (Liu et al., 2020). Copyright 2020, Elsevier. **(E)** TEM image of core-shell nanofibers with BMP-2 aqueous solution as the core while the blend of SP600125 and gelatin as the shell. Reproduced with permission (Liu et al., 2020). Copyright 2020, Elsevier.

In the aspect of structural modification, recent studies are paying emphasis on developing core-shell nanofibers using the coaxial electrospinning technique (Yang et al., 2020). To date, the core-shell nanofibers, serving as a class of novel drug delivery carriers, have been widely applied in oral diseases (Xu et al., 2006; Yang et al., 2015; Xia et al., 2018; Wen et al., 2020). In one typical study, drug-loaded micelles were encapsulated into electrospun fibers to realize the controlled release of the payloads. Usually, the drug-loaded micelles were expected to be incorporated into the core of fibers. In this way, the shell layer not only enhanced the mechanical strength of nanofibers but also functionalized as an ideal platform for drug storage and delivery. In one study, two types of poly (ethyleneglycol)-block-polycaprolactone (PEG-b-PCL) micelles containing SP600125 (c-Jun N-terminal kinase inhibitor) and SB203580 (p38 MAP kinase inhibitor), respectively, were encapsulated into gelatin fibers (Figure 2A) (Wang Y. et al., 2019). In this way, drugs were released from the microspheres and then went across the shell to achieve a programmatic release. Such an approach can also be used for dental pulp therapy. A dental pulp restoration drug, dexamethasone (DEX), has been loaded into the hydrophobic cavity of β-cyclodextrin (β-CD) inclusion complex and then mixed with PLGA solution (Figures 2B,C) (Daghrery et al., 2020). The DEX is finally trapped in PLGA shell after electrospinning process. The release profiles proved the release of DEX with a high efficiency.

Through the use of coaxial electrospinning, core-shell fibers can also be simply fabricated with the use of a rich variety of materials and payloads. For example, an anti-inflammation drug SP600125 was mixed with gelatin as the shell, while bone morphogenetic protein 2 (BMP-2) aqueous solution was selected as the core solution to generate drug-loaded, core-shell nanofibers (Figures 2D,E) (Liu et al., 2020). Such kind of core-shell structure allowed the anti-inflammatory and osteogenic drugs to be released in the expected order to exert their respective therapeutic effects. Taken together, nanofibers with core-shell structures have been a class of ideal candidates for drug delivery in oral disease therapy.

### Multi-Layered Membranes

**FIGURE 3:**
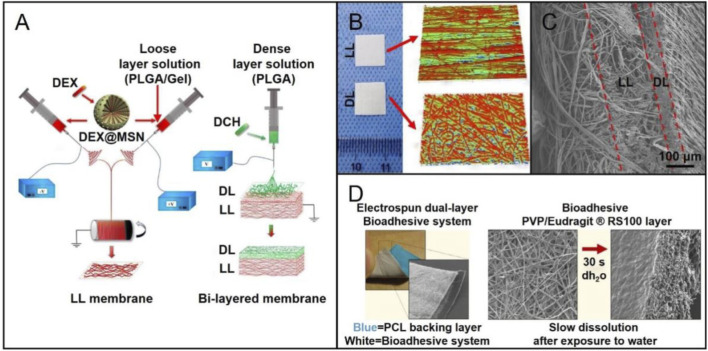
**(A)** Schematic diagram showing the preparation of a bi-layered GBR membrane: fabrication of the PLGA/gelatin nanofibrous membrane (loose layer) using the conjugated electrospinning method with a customized rotating collector **(left)**; deposition of the PLGA nanofibers (dense layer) on the loose layer by the traditional electrospinning to form the bi-layered GBR membrane **(right)**. Reproduced with permission (Lian et al., 2019). Copyright 2019, Elsevier. **(B)** Macroscopic and surface topography appearances of the dense layer (DL) and loose layer (LL). Reproduced with permission (Lian et al., 2019). Copyright 2019, Elsevier. **(C)** Cross-sectional SEM image of the bi-layered membrane. Reproduced with permission (Lian et al., 2019). Copyright 2019, Elsevier. **(D)** Photograph of the dual-layer bioadhesive system, and the SEM image showing the PCL fibers in the backing layer **(left)**. SEM images of the bioadhesive layer made of a blend of poly (vinylpyrrolidone) (PVP) and Eudragit RS100 (RS100) before and after dissolved in water. Reproduced with permission (Santocildes-Romero et al., 2017). Copyright 2017, American Chemical Society.

Compared with traditional two-dimensional (2D) nanofiber mats, multilayered, three-dimensional (3D) nanofibrous scaffolds have unique advantages in regulating cell behavior and promoting tissue regeneration (Greiner and Wendorff, 2007; Liao et al., 2005; Bottino et al., 2011; Joshi et al., 2015; Qian et al., 2018; Rezk et al., 2018). Researchers have found that the combination of layers with different porosity can better guide bone and cartilage regeneration. Moreover, the scaffold will provide enough space to deliver drugs or bioactive factors, which can further promote the regeneration of injured site (Ionescu et al., 2010). In particular, two or more kinds of drugs loaded in the different layers can achieve synergistic release of the drugs. Owing to these specific advantages, a wide range of multilayered scaffolds have been developed. For example, a bi-layered GBR membrane was obtained *via* a two-step electrospinning method (Figure 3A). The loose layer consisting of PLGA/gelatin (PLGA/Gel) fiber was firstly prepared by the conjugated electrospinning technique (Figures 3B,C) (Lian et al., 2019). Then, a dense layer composed of PLGA fiber was fabricated by the traditional electrospinning technique. In order to enhance the osteogenesis capacity, DEX-loaded mesoporous silica nanoparticles (DEX@MSNs) were added into the loose layer. Meantime, doxycycline hyclate was incorporated in the dense layer, which could stop cell infiltration and inhibit bacterial infection. In this way, the bi-layered scaffold enhanced bone formation and regeneration due to its structure and the release of payloads.

In addition to the bi-layered scaffolds, multilayer scaffolds have also received more and more attentions for use in the regeneration of injured tissues. In one study, a tri-layered scaffold was constructed with both sustainable drug-release system and mineralization function to promote bone formation (Rezk et al., 2018). In this case, the superficial layer made of poly (vinyl alcohol)-poly (vinyl acetate) (PVA-PVAc) provided an appropriate platform for releasing simvastatin (SIM), which had been proved to cure osteoporosis, induce osteogenesis differentiation, and promote bone formation. In addition, the biomineralization function provided by PCL-cellulose acetate-β-tri calcium phosphate (PCL-CA-β-tcp) middle layer could further enhance bone formation. In this case, β-tcp was effective in assembling mineral contents on the surface of electrospun nanofibers. PCL nanofibers were selected as the fundamental layer to guarantee the mechanical strength of the scaffolds. When curing the oral mucosal lesions, multilayered nanofiber membranes also show good possibility. In one study, a mucoadhesive membrane was made up of two nanofiber layers: one layer was prepared by adding particles of dextran or poly (ethylene oxide) (PEO) to PVP/Eudragit RS100 fibers (Figure 3D) (Santocildes-Romero et al., 2017). Then, PCL nanofibers were serving as the backing layer to construct the dual-layered bioadhesive system. When implanted such scaffolds into oral mucosal lesions of porcine, the mucoadhesive property was improved, and the adhesion time was extended. In conclusion, the combination of growth factors, nanoparticles, and drug and/or cell delivery with advanced multilayered scaffolds holds great capability to facilitate the curing of oral diseases.

### Composite Scaffolds

**FIGURE 4:**
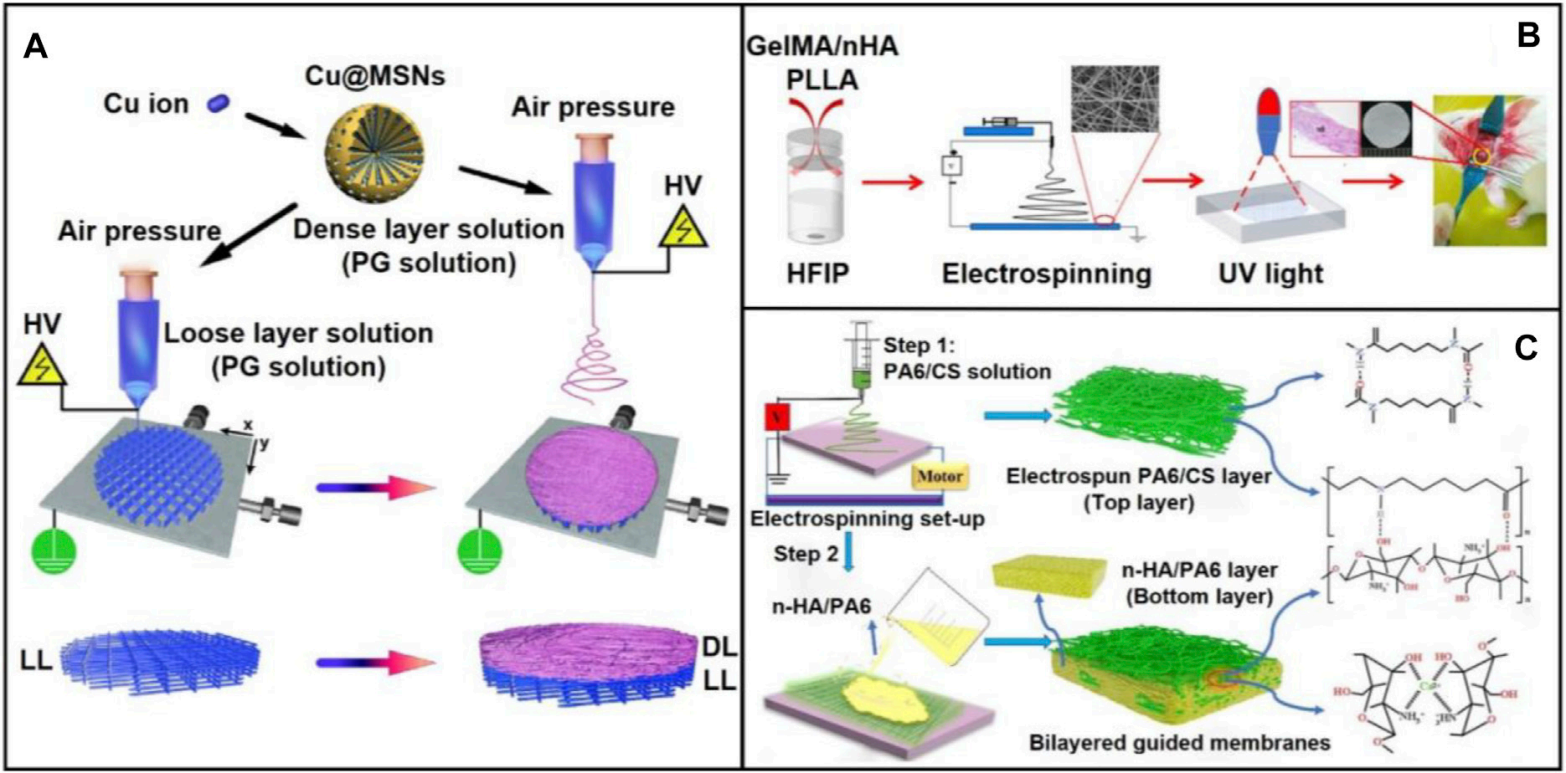
**(A)** Schematic diagram showing the preparation process and application scenario of the bi-layered electrowritten PLGA/gelatin-Cu@MSNs scaffold. The bi-layered PLGA/gelatin-Cu@MSNs GBR scaffold was fabricated using a single SEW printer through a facile two-step procedure. The bi-layered composite scaffold, consisting of a loose and porous SEW layer and a dense and compact SES layer, was prepared by depositing dense layer nanofibers onto the preprinted loose layer scaffold. Reproduced with permission (Lian et al., 2020). Copyright 2020, Acta Materialia Inc. (Elsevier). **(B)** Schematic illustration showing the fabrication a biomimetic membrane consisting of GelMA/n-HA/PLA and its use for enhanced bone regeneration. Reproduced with permission (Li et al., 2020). Copyright 2020, American Chemical Society. **(C)** Schematic diagram showing the preparation of bilayered guided membranes with polyamide-6/chitosan nanofiber as the top layer while the n-HA/polyamide-6 film as the bottom layer. Reproduced with permission (Niu et al., 2021). Copyright 2021, Elsevier.

Due to the complicated repair or regeneration process of injured tissues, one should expect to design a scaffold holding appropriate degradation ability, bioactivity, biocompatibility, and suitable mechanical strengths. To this end, composite scaffolds would be a best choice to achieve a balance between the different properties (Jose et al., 2009; Gugutkov et al., 2017; Wang B. et al., 2018). As reported, composite scaffolds are particularly suitable for the repair of periodontium complex including cementum, periodontal ligament, and alveolar bone. In one study, a functional bilayered scaffold including a dense layer and a loose layer was fabricated *via* solution electrospinning writing (SEW) and electrospinning techniques (Figure 4A) (Lian et al., 2020). During the fabrication, copper-loaded MSNs (Cu@MSNs) were mixed with PLGA and gelatin solution. The dense layer made of PLGA/gelatin fibers could serve as a barrier to prevent the disturbance of non-osteoblasts during osteogenic differentiation. The loose and porous layer which obtained *via* SEW technique induced and promoted bone growth and regeneration.

Although a wide range of electrospun fibers made of single or mixed polymers could provide sufficient mechanical strength and space for cell adhesion, they often lacked osteogenic and antibacterial properties, limiting their capability of repairing bulk damaged bone tissue. In this regard, composite scaffolds designed to mimic the components and/or structures of native extracellular matrix (ECM) should be paid more attention for the precision repair of oral defects. For instance, a hydrogel-nanofiber composite was designed by co-electrospinning the gelatin methacrylate (GelMA)/n-HA complex and PLA (Figure 4B) (Li et al., 2020). Using such a composite scaffold, good mechanical property, biocompatibility, and bone regeneration capacity were achieved within one scaffold. Osteogenic differentiation was also significantly enhanced in rat critical defects model. In another study, bilayered guided membranes with polyamide-6/chitosan fibers as the top layer while the n-HA/polyamide-6 film as the bottom layer were fabricated for guided bone regeneration (Figure 4C) (Niu et al., 2021). This two-step approach mainly involved the use of electrospinning technique for preparing the top layer and solvent casting for producing the bottom layer. In this design, polyamide-6 acted as a collagen matrix that endowed the composite scaffold with excellent bioactivity. To maintain the mechanical strength, polyamide-6/chitosan nanofibers was used for the reinforcement. In particular, owing to the composition of n-HA nanoparticles, osteoconductivity was highly promoted.

Compared with the commonly-used surface modification approach such as physical absorption, chemical modification, grafting, and plasma treatment, the mussel-inspired polydopamine (PDA) coatings technique is a promising alternative that could allow for the modification of different functional molecules. In one typical study, the silver nanoparticles (AgNPs) were modified on the surface of PDA-coated electrospun PLGA/PCL nanofibers to obtain the antibacterial capability (Qian et al., 2019). In order to enhance the osteogenesis capability, type I collagen was further immobilized onto the AgNPs-PLGA/PCL nanofibers by immersing the obtained scaffold into the 2% collagen solution. Using this strategy, the collagen-modified AgNPs-PLGA/PCL nanofibers could simultaneously prevent bacterial infection and promote bone regeneration.

## Engineering Nanofibers With Biofunctions

### Biological Effects Provided by Bioactive Proteins and Other Components

**FIGURE 5:**
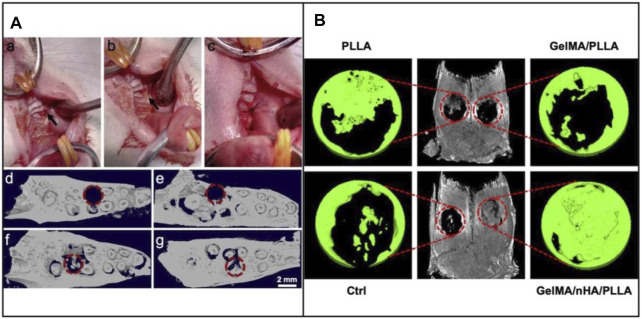
**(A)** Intraoperative image of the critical-sized defect created in rat maxillae (upper jaw) after extraction of the first molar tooth. (a) Defect drilled into the upper jaw bone (indicated by arrow), (b) Filling of defect with mineralized nanofiber graft, and (c) Suturing of the tissue around the defect to hold the graft in place. Representative 3D reconstructions of coronal view micro-CT images of the defect, (d) Immediately after tooth extraction and defect creation, (e) Unfilled defect/control after 4 weeks, (f) Defect filled with mineralized PLGA/collagen/gelatin nanofiber fragments, and (g) Defects filled with BMP-2 loaded mineralized PLGA/collagen/gelatin nanofiber fragments. The dashed circles indicate the defect regions. Reproduced with permission (Boda et al., 2019). Copyright 2019, Acta Materialia Inc. (Elsevier). **(B)** *In vivo* assessment of bone regeneration capability. 3D reconstructed micro-CT images of rat cranial bone and magnified images of bone defects at 12 weeks following surgery. The red circles indicate the created critical-sized 5 mm defects. Reproduced with permission (Li et al., 2020). Copyright 2020, American Chemical Society.

In addition to the topographic cues provided by the structural changes of the underneath fibers, the tissue repair performance can also be greatly improved by the biological cues offered by bioactive proteins or other functional components (Dong et al., 2009; Wang S. et al., 2018; Li et al., 2019; Seal et al., 2020). For example, BMP-2, an osteoinductive polypeptide, was integrated into mineralized PLGA/collagen/gelatin nanofiber fragments for regenerating defected alveolar bone (Figure 5A) (Boda et al., 2019). An *in vivo* alveolar bone defect model indicated the effect of calcium-binding BMP-2 peptides on the repair of damaged alveolar bone. Scaffolds loaded with functional nanoparticles have also been recognized as promising alternatives for the repair of oral defects. In one study, cerium oxide nanoparticles (CeO_2_ NPs), which could provide anti-inflammation, anti-oxidant, and anti-bacterial capabilities, were encapsulated into electrospun PCL/gelatin fibers (Ren et al., 2021). The CeO_2_ NPs-loaded fibers promoted better differentiation of human periodontal ligament stem cells to osteoblasts than that using the PCL/gelatin fibers. *In vivo* results indicated the improved bone regeneration using the CeO_2_ NPs-loaded fibers. In another study, n-HA nanoparticles were incorporated in the fibers made of PLA and GelMA during electrospinning (Li et al., 2020). The incorporation of n-HA and GelMA successfully enhanced the bone regeneration, mainly attributed to the biomimetic architecture to native bone ECMs and the addition of mineralized components (Figure 5B).

Bacterial infection, which usually originates from wound oral fluids, has also been a critical problem when treating oral diseases. To address this issue, the development of anti-microbial nanofibers has been an effective way. In one typical study, an anti-microbial peptides-loaded multifunctional membrane was fabricated by electrospinning and electrospray techniques (He et al., 2018). In this study, the PLGA microspheres loaded with AMPs were deposited on the surface of electrospun nanofibers made of gelatin and chitosan, contributing to the sustained release of AMPs for a longer time. The antibacterial results showed obviously antibacterial activity against *S. aureus* and *E. coli*, holding potential of protecting the oral wound from being infected.

### Cell Delivery

**FIGURE 6:**
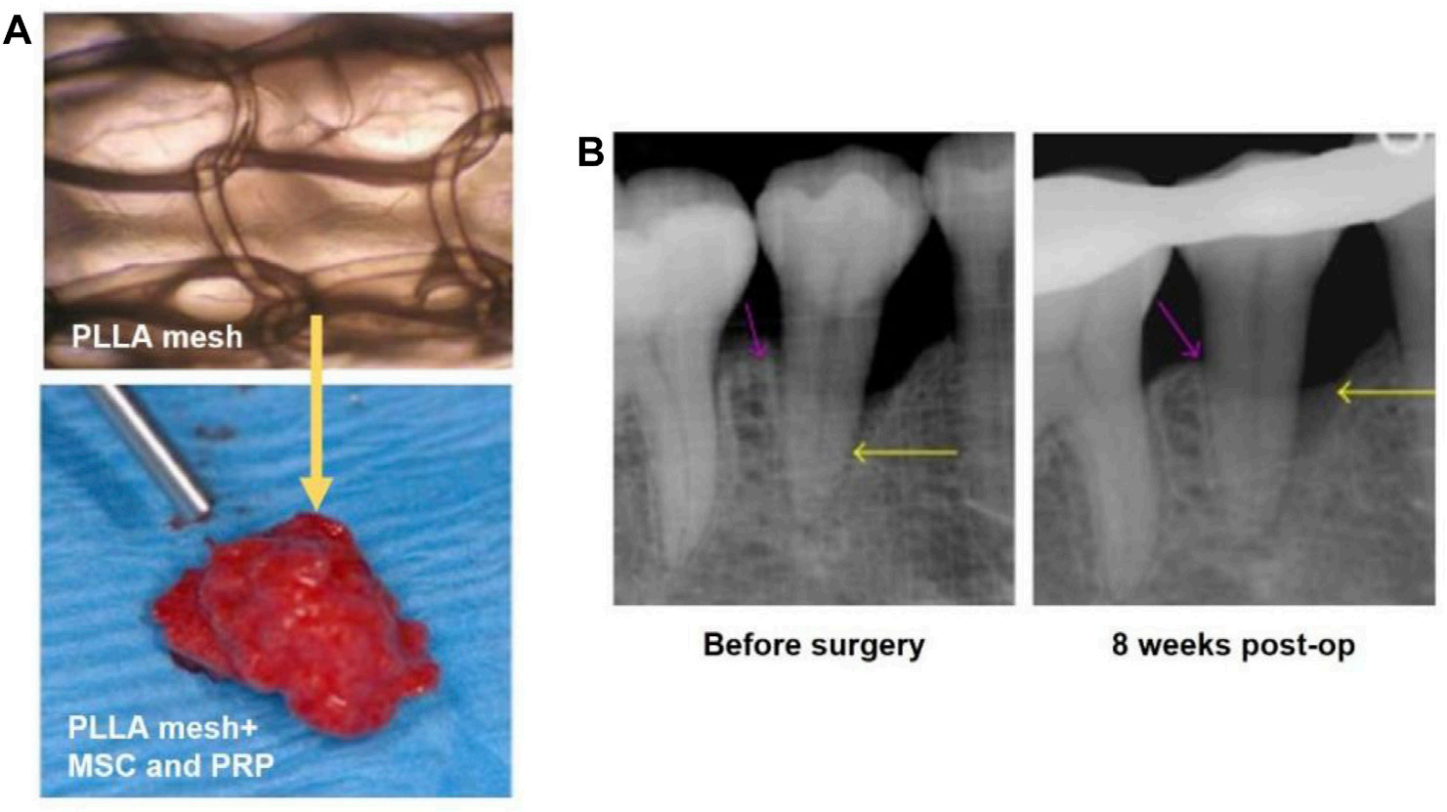
**(A)** The basket-shaped PLA mesh (top). The BMSCs and PRP were perfused in the PLA mesh for cell delivery (bottom). Reproduced with permission (Baba et al., 2016). Copyright 2016, Shunsuke Baba et al. **(B)** X-ray images showing the depth of the intrabony defects before and at 8 weeks post surgery as indicated by the yellow arrows. Reproduced with permission (Baba et al., 2016). Copyright 2016, Shunsuke Baba et al.

Cell delivery and treatment strategy has exhibited great therapeutic effect and potential in tissue engineering (Mooney and Vandenburgh, 2008) (Facklam et al., 2020). For example, immune cells or stem cells can be transported to the damaged areas and differentiate to cells or tissues with recovered functions (Rai et al., 2015). Stem cells that are normally used in oral disease therapy include embryonic stem cell, bone mesenchymal stem cell (BMSC_S_), and adipose stem cells (ADSCs) (Rai et al., 2015) (Baba et al., 2016). In one typical study, a 3D woven fabric composed of basket-shaped PLA fibers (Figure 6A) was developed to load BMSCs and platelet-rich-plasma (PRP). In a typical process, the mixture of BMSCs and PRP was prepared with the combination of human thrombin in 10% calcium chloride. Then, the mixture was perfused in the PLA scaffold (Figure 6B). With the addition of PRP, the proliferation and differentiation of BMSCs were improved, together with the improvement of the biofunctionability of PLA scaffold. The 3-year clinical trials proved the safety and efficacy of stem cell-based therapy. Most important, the BMSCs/PRP-loaded scaffold significantly promoted the regeneration of periodontal tissues.

In addition to BMSCs, ADSCs also exhibit great potential in cell therapy (Nakahara, 2006). For example, ADSCs show more advantages such as the simplicity of extraction as well as the robust growth and reproduction ability (Zuk et al., 2002). In one study, a ADSCs-loaded, PLGA scaffold was fabricated for the treatment of periodontitis (Akita et al., 2014). In this case, the PLGA scaffold was produced using solvent casting-particulate leaching technique. Then, the ADSCs were seeded on the PLGA scaffold, contributing to the significant improvement of periodontal tissue regeneration relative to the case involving the use of blank PLGA scaffold. As there are few studies reporting the use of electrospun fibers for cell therapy to treat oral diseases, we expect the rational design of fiber materials to serve as the highly-efficient carriers to maintain the cell activity as well as the effective delivery of stem or repairable cells for the regeneration of oral tissues.

### Drug Delivery

Electrospun fibers have shown great potential to functionalize as carriers to incorporate drugs and realized the controlled release of the payloads. To date, a variety of drugs have been encapsulated into different electrospun fibers to endow the scaffolds with biofunctions, for example, anti-inflammatory, antibiotic, osteogenic induction, and anticancer (Yoo et al., 2009; Zamani et al., 2010; Karthikeyan et al., 2012; Qiu et al., 2013; Jiang et al., 2014; Zhao et al., 2015; Yuan et al., 2016; Huang et al., 2019; Clitherow et al., 2020). In particular, many recent studies have suggested that the drug-loaded nanofibers possess synergistic therapeutic effect during the cure of oral diseases (Wang et al., 2020; Rade et al., 2021). In one study, DEX was embedded in a hydrophobic central cavity of CD. Then, the DEX-CD complex was mixed with PLGA solution for electrospinning to obtain the DEX-CD@PLGA fibers. Such fibers induced the osteogenic differentiation and attenuated the inflammatory response when repairing the dental pulp tissues (Daghrery et al., 2020). In another example, electrospun PEO/PLA fibers that could locally deliver Lipoxin A_4_ was developed to improve the proliferation and migration of dental stem cells in order to facilitate the repair of periodontal tissues (Cianci et al., 2016).

**FIGURE 7:**
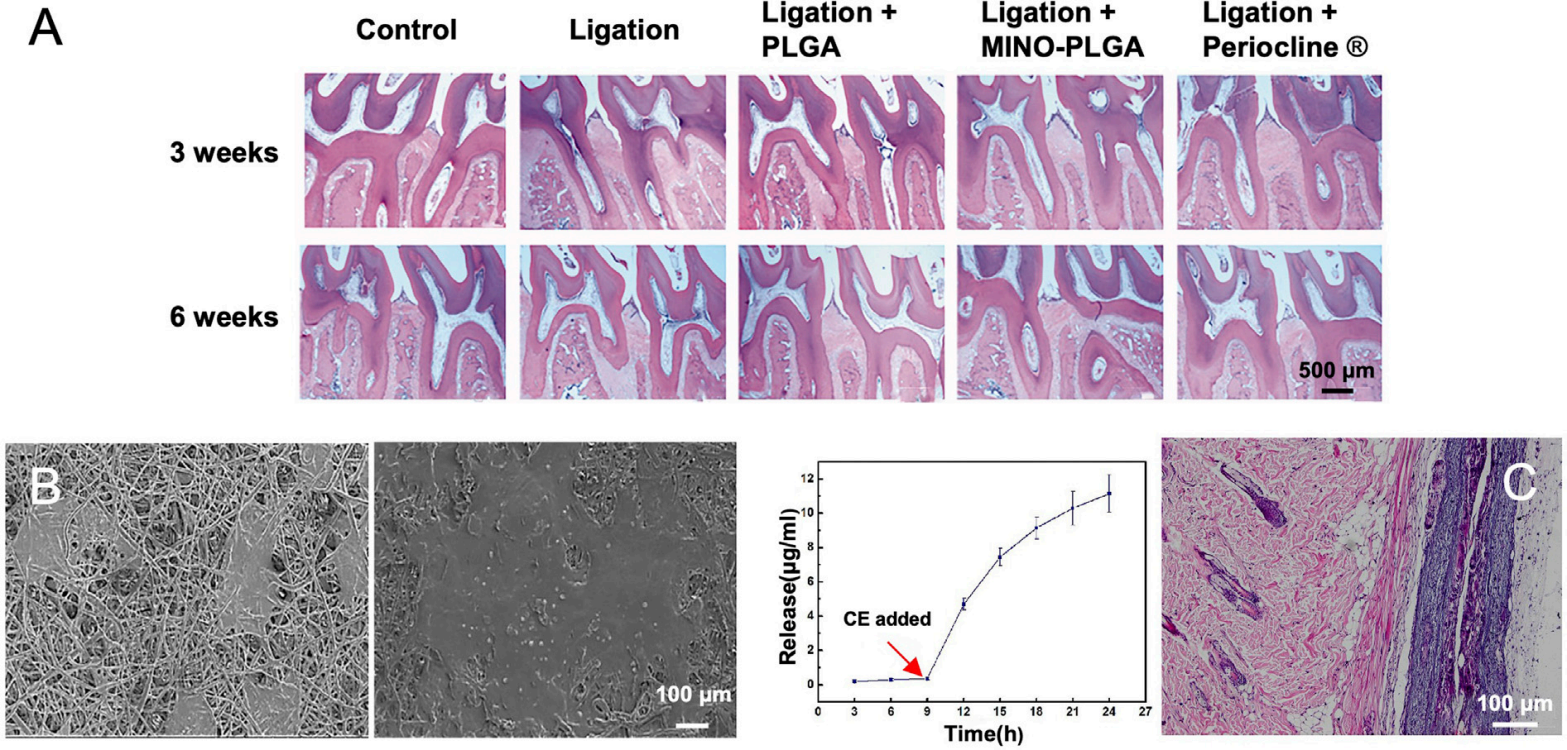
**(A)** Histological observation (H&E stain) of maxillary periodontal tissues at 3 and 6 weeks post treatment. Reproduced with permission (Ma et al., 2020). Copyright 2020, Creative Commons Attribution License. **(B)** SEM images showing the morphology of MC3T3-E1 cells after culture on the surfaces of multilayered nanofibers scaffolds containing β-tcp and SIM for 1 and 3 days, respectively. Reproduced with permission (Rezk et al., 2018). Copyright 2018, Elsevier. **(C)** Pro-MNA release profile during a period of 24 h with the addition of cholesterol esterase at the ninth hour **(left)**. Histological micrograph showing H&E staining at 8 weeks post implantation of the Pro-MNA-loaded scaffold **(right)**. Reproduced with permission (He et al., 2020). Copyright 2020, Elsevier.

As a broad-spectrum antimicrobial drug, minocycline (MINO) was successfully added into the PLGA solution for preparing antibiotic electrospun nanofibers (Ma et al., 2020). *In vitro* and *in vivo* results indicated that the sustained release of MINO could reduce inflammatory responses and improve bone formation when treating periodontitis (Figure 7A). Owing to the relatively high specific surface area and adjustable porosity, electrospun fibers offered sufficient space and versatility for drug delivery and release. Multilayered nanofiber membranes are also a class of promising alternatives for drug delivery and oral tissue regeneration. In one study, a multilayered scaffold with SIM-loaded PVA-PVAc nanofibers as the superficial layer, PCL-CA-β-tcp fibers as the middle layer, and PCL fiber serving as the backing layer was developed for the enhancement of osteogenesis and mineralization (Rezk et al., 2018). Owing to this specifically structural design and the controlled release of SIM, the mechanical property, biocompatibility, as well as the promotion of osteogenesis were all improved (Figure 7B). In another example, pro-metronidazole (Pro-MNA), serving as an antibacterial drug, was loaded into gelatin fibers to directly contact with the defect sites for the purpose of protecting bacterial infection (He et al., 2020). As shown in Figure 7C, the release profile also showed that Pro-MNA was infection-responsively released over a duration of 24 h. Hematoxylin and eosin (H&E) staining results revealed that few mononuclear cells and fibroblasts were observed at 8 weeks post implantation of the Pro-MNA-loaded scaffold.

## Translational Application of Electrospun Fibers for Oral Disease Treatment

### Periodontium Regeneration

Periodontitis is a chronic inflammation mainly caused by local factors, which can cause the destruction of periodontal tissues and eventually lead to tooth loss, affecting about 15% of adults in the world (Abdelaziz et al., 2021). The periodontium consists of gingiva, alveolar bone, periodontal ligament (PDL), and cementum. Among others, gingiva and PDL are fibrous tissues that can fix the cementum of root to the adjacent alveolar bone. Cementum and alveolar bone are mineralized tissues, which surround and support the teeth. At present, it is expected to repair the periodontal tissue by reconstructing its original hierarchical structure, including the formation of new cementum, alveolar bone, and PDL (Ripamonti and Petit, 2009).

Trauma, tumor resection, and periodontal infection can all lead to large defects of periodontal tissues in oral cavity. Guided tissue regeneration (GTR) and GBR are the two main methods for the regeneration and repair of periodontal tissue defects. The GTR and GBR membranes were usually required to be biocompatible and could be integrated into surrounding tissues without causing inflammation response. As nanofibrous scaffolds could emulate the native ECM, GTR/GBR membranes made of nanofibers are capable of recruiting stem cells and progenitor cells located in adjacent PDL, alveolar bone, and blood. These cells can differentiate into fibroblasts, osteoblasts, and cementoblasts to form new PDL, bone, and cementum (Larsson et al., 2016).

**FIGURE 8:**
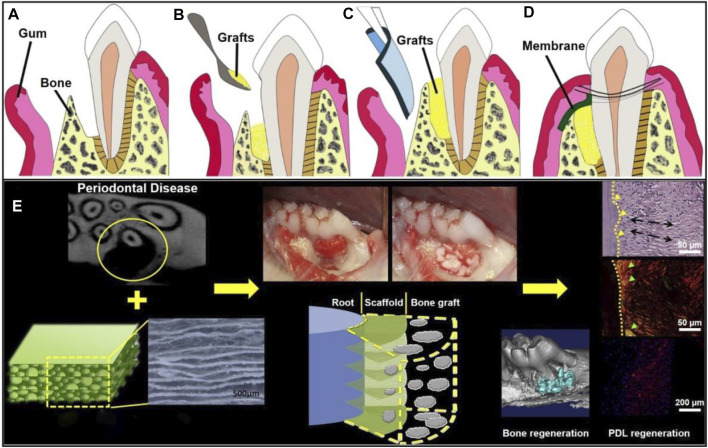
Schematic illustration of a GTR membrane combined with bone graft therapy for periodontal regeneration. **(A)** Periodontal defect with loss of PDL and alveolar bone. Reproduced with permission (Chen et al., 2010). Copyright 2010, Elsevier. **(B)** Bone grafts at the defected site. Reproduced with permission (Chen et al., 2010). Copyright 2010, Elsevier. **(C)** GTR/GBR membrane covered on the grafts. Reproduced with permission (Chen et al., 2010). Copyright 2010, Elsevier. **(D)** Sewing for wound closure. Reproduced with permission (Chen et al., 2010). Copyright 2010, Elsevier. **(E)** Organized PDL regeneration with the use of a composite scaffold made of PCL-PEG nanofibers and chitosan. Reproduced with permission (Jiang et al., 2015). Copyright 2015, Elsevier.

Nanofibrous GTR and GBR membranes could be designed with the use of a wide range of synthetic polymers and/or natural biomaterials by electrospinning. Such kind of membranes had excellent biocompatibility, biodegradability, bone conductivity and bone induction, which could regulate cell attachment, proliferation, and differentiation to promote periodontal regeneration (Zamani et al., 2010). By adding different polymers and various additives (such as active bioceramics, growth factors, proteins, and drugs) to electrospun nanofibers, different requirements regarding the periodontal regeneration can be met (Zhuang et al., 2019). GTR is normally combined with bone graft technology to treat periodontal defects, in which the membrane is used to help stabilize bone transplantation and replace gingival tissue. As gingival tissue grows much faster than bone, the membranes are required to prevent the growth and displacement of gingival tissue before bone mature (Figure 8A–D) (Chen et al., 2010). In one study, electrospun PCL-PEG nanofibers were combined with porous chitosan scaffold to realize the directional regeneration of PDL. It was found that arranged PDL-like tissues were regenerated at the defect site in a rat model after 2 months, which were very close to the original PDL. The results also indicated that the scaffold could enhance the infiltration, viability, and periosteal protein expression of rat BMSCs, leading to a more orderly arrangement of regenerated PDL (Figure 8E) (Jiang et al., 2015).

In addition to synthetic and natural materials, inorganic components such as ceramics, calcium phosphorus-based, calcium silicon-based, carbon-based, oxide and metal components can also be blended with polymers to prepare composite nanofiber membranes to mimic the native bone tissues. For example, type I collagen and n-HA represent promising alternatives to endow the PCL scaffold with good biocompatibility and bone induction capability in periodontal tissue engineering (Wu et al., 2014). In another study, a bilayered composite membrane made of PLGA/multiwall carbon nanotubes (MWNTs)/bacterial cellulose was developed to facilitate the repair of maxillary canine periodontal tissue defect in beagle dogs (Zhang et al., 2018). Bioactive glass can also be blended to improve the bone conductivity and biophysical-chemical properties of the scaffolds to simulate natural inorganic bone components (Qasim et al., 2015). As a typical oxide component, ZnO has been used to electrospin with PCL to obtain excellent bone regeneration and antibacterial capabilities, which made the scaffold a promising candidate for regulating inflammatory environment and promoting bone formation in periodontal regeneration (Nasajpour et al., 2018). In addition to the selection of different types of fiber materials, drugs, growth factors, and proteins can also be added to electrospun nanofibers to obtain the corresponding biological functions. In this way, anti-inflammatory effect as well as periodontal regeneration can also be improved (Zhuang et al., 2019). Taken together, these results indicate that functionalized electrospun nanofiber membranes show broad application prospects in periodontal tissue regeneration (Pajoumshariati et al., 2018).

### Mandibular Repair

Mandible, as the main supporting structure of bone, plays an important role in maintaining the contour and shape of face. Mandible has a vital impact on our daily activities, such as chewing and speaking. Current studies have indicated that tumors, infections, functional atrophy, congenital diseases, periodontitis, and iatrogenic injuries are the primary reasons to mandibular damage (Rubio-Palau et al., 2016). Patients with mandibular damage usually go through facial pain and changes in face appearance. They also bear a heavy psychological burden and even suffer from mental health problems such as depression (Malá et al., 2015). When considering the repair of mandibular, it really depends on the size of the defects. Normally, a small-size defect requires no special treatment. However, when the defect region is large, bone gratfing is necessary. To date, the most commonly used treatment methods are allograft and xenogeneic transplantation. However, these two methods may cause problems such as immune rejection and foreign body response. Hence, there is an urgent need for the development of new repair methods for the current clinical treatment.

With the rapid development of engineering strategies to generate biomedical materials, scaffolds with specifically designed structures and biological properties have emerged for mandibular defect repair. The biocompatibility, mechanical properties, and other superior functions of biomedical materials determine their great potential in repairing mandibular defects. (Wang L. et al., 2019; Zhang et al., 2019). For example, a composite nanofiber scaffold composed of polyaniline and polyvinylidene fluoride was prepared to enhance stem cell viability and adhesion by utilizing a pulsed electromagnetic field (Mirzaei et al., 2019). Results showed that the scaffold promoted ell attachment under the conductivity due to the addition of polyaniline in an extremely low frequency pulsed electromagnetic field. In particular, the scaffold enhanced the osteogenic differentiation of stem cells derived from dental pulp for mandible regeneration.

### Oral Mucosa Repair

It is well known that oral mucosal defects usually refer to the ulcers, cuts, and abrasions of oral cavity, as well as lesions caused by recurrent aphthous stomatitis or oral lichen planus (Akintoye and Greenberg, 2005). In addition, for cancer patients, oral mucositis often occurs during chemotherapy and radiotherapy treatments, and the uptake of anti-cancer drugs would also lead to or even aggravate the defects (Sonis, 2009). After oral mucosal injury, there is a complex process from injury to complete healing, involving primary injury reaction, signal amplification, ulceration, and healing. Therefore, it is necessary to take actions at different stages of healing to promote the repair of oral mucosal tissues (Sonis, 2009). At present, the inhabitation of inflammatory response using steroids is the most common immune-modulating approach. However, the lack of specific ointments and creams containing steroids and their analogues limits the further application of this immune-modulating method. Most ointments and creams have very poor adhesion to the surface of the oral mucosa. The agitation of tongue and the chewing of teeth facilitate ointment and cream fall off, making the contact time between the medication with the wound short, and the treatment is ineffective. Besides, the steroids products designed for patients with dermatosis are often used for repairing oral mucosa defects, whose doses and precautions are not fully appropriate for patients with mucositis. As such, there is an exigent requirement to invent new medical materials that can be applied for local administration and own well hydrophilicity and adsorption properties.

**FIGURE 9:**
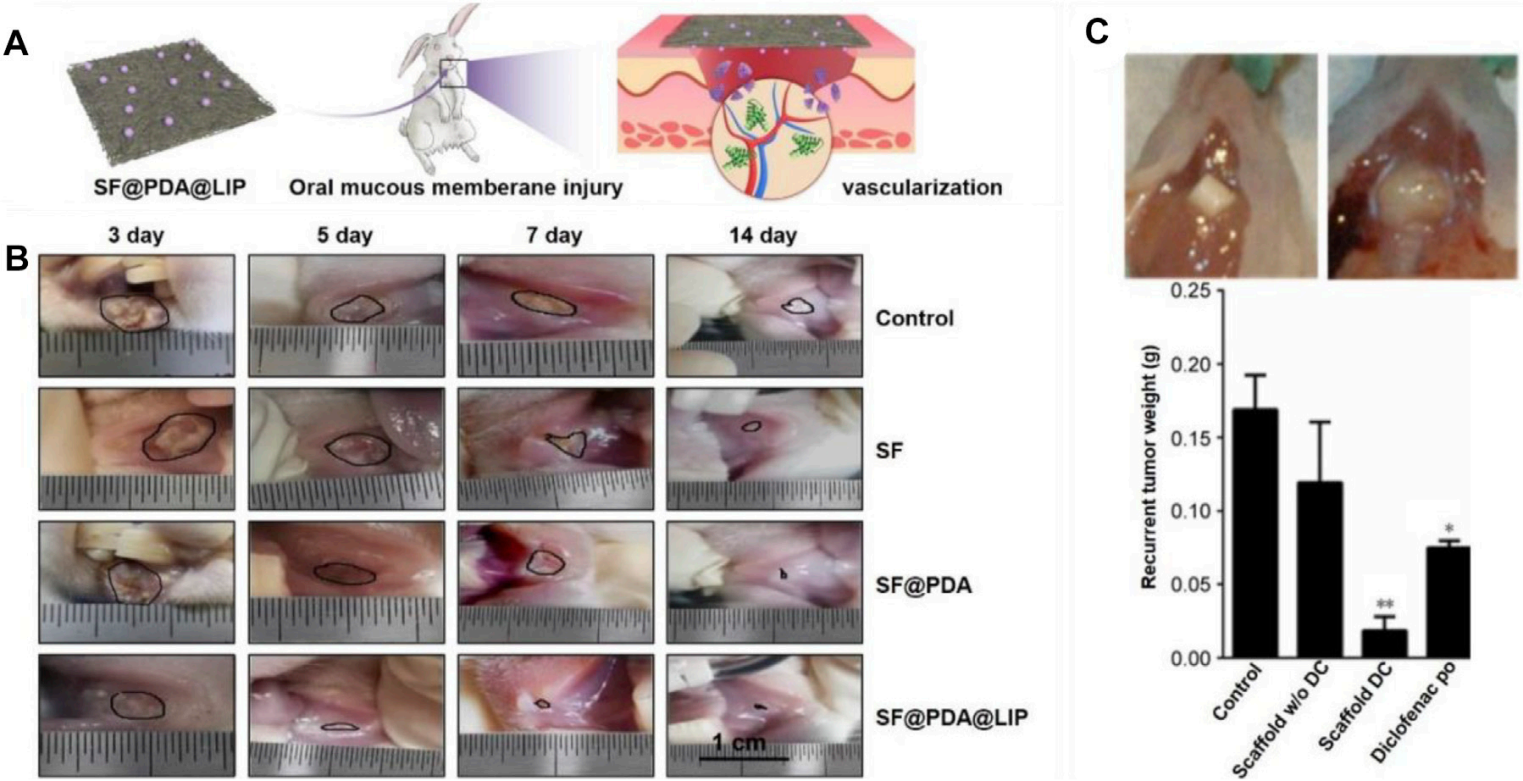
**(A)** Silk fibroin fibers deposited with leptin-loaded NH_2_-LIPs were used to cover oral mucous membrane injury to promote angiogenesis. Reproduced with permission (Qian et al., 2020). Copyright 2020, Creative Commons Attribution. **(B)** Photographs showing the healing of injuries as marked by black lines. Reproduced with permission (Qian et al., 2020). Copyright 2020, Creative Commons Attribution. **(C)** Effect of the local release of diclofenac on recurrent tumor weight. Representative photographs showing the recurrent tumor weight was lower when treated by the diclofenac-loaded scaffold at 7 weeks post surgery **(top left)**. The largest recurrent tumor was observed in the untreated group **(top right)**. Weight distribution of recurrent tumors in the different experimental groups. The lowest average weight was observed in mice implanted with diclofenac-releasing scaffolds. *p < 0.05, **p < 0.01 (bottom). Reproduced with permission (Will et al., 2016). Copyright 2020, Dovepress.

Tissue engineering strategy has made it possible for *in situ* drug deliver and design scaffolds with the above-mentioned properties (Karthikeyan et al., 2012; Gajdziok et al., 2015; Clitherow et al., 2020). Herein, we report the engineering of electrospun nanofibers applied in oral mucosa repair. In one study, a electrospinning solution was prepaed using 97% (v/v) ethanol containing RS100 and PVP (Santocildes-Romero et al., 2017). To further enhance the mucoadhesive properties, dextran and PEO were uniformly distributed into the solution before electrospinning. The mucoadhesive membrane was implanted into the oral cavity of porcine, and *in vitro* results proved the strong adhesion performance and great potential in oral mucosal repair. In addition to the adhesion performance, vascularization also plays an important role in oral mucosa repair. In one typical study, leptin-loaded liposomes were modified with amino groups (NH_2_-LIPs) and then deposited on the surfaces of silk fibroin fibers (Figure 9A) (Qian et al., 2020). The conjugated NH_2_-LIP-wrapped leptin could be locally released, thereby enhancing the vascularization and wound healing of the injured mucosa area in a rabbit model (Figure 9B). Another study also reported the utilization of drug-loaded electrospun fibers with optimized release behavior and ideal degradation time for treating oral mucosa defects. In this study, Eudragit L 100 and human growth hormone (hGH) were used as raw materials to manufacture the fiber mats *via* a co-electrospinning process. Then, the fibrous mat was coated with a chitosan layer using a dip-coating method and implanted into the animals with oral mucositis (Choi et al., 2016). With the release of hGH, it would be adsorbed on the surface of chitosan layer due to the ionic reaction, thus promoting the viability and proliferation of human dermal fibroblasts. After employing the mat in the oral ulcers of dogs, it was obviously that the oral ulcers decreased, and epithelium was significantly regenerated in comparison to the mat without loading of hGH.

### Oral Cancer Therapy

Cancer is often referred as the uncontrolled overgrowth of cells and metastasis to a variety of organs, which is one of the main reasons of death (Nosrati et al., 2018). Among others, oral cancer, as the sixth most common malignant tumor, is clinically difficult to treat, causing great physical and mental torture to patients (Aparna et al., 2015). The current clinical treatment methods include surgery, radiotherapy, chemotherapy, and the use of anti-cancer drugs. Nevertheless, these treatments are usually accompanied by certain side effects. Taking the treatment of oral squamous cell carcinoma (OSCC) as an example, it is the most common oral cancer accompanied with high morbidity and mortality. The modern surgery and treatment strategies such as cisplatin, cetuximab, and taxanes still have their own limitations (Borges et al., 2017). Current treatment approaches still possess low survival rates and prominent toxicities. Therefore, there is an urgent need for the development of more effective and appropriate treatment strategies for curing OSCC and other cell carcinomas. The emergence of biomedical materials has provided new avenues for *in-situ* treating oral cancers, some of which have been utilized to prevent tumor recurrence (Jiang et al., 2014).

In one typical study, electrospun poly (D,L-lactide-co-glycolide) nanofiber scaffolds were designed with sustained-release of diclofenac to inhibit oral tumor growth (Will et al., 2016). *In vitro* cell evaluation indicated that the poly (D,L-lactide-co-glycolide) nanofiber scaffold showed highly effective impact on killing squamous cell carcinoma cells. In particular, *in vivo* results suggested that the locally released diclofenac could significantly decrease the tumor recurrence rate relative to the cases involving the use of nanofiber scaffolds without the release of diclofenac, taking diclofenac orally, and the control group without any treatment. Thirty-three percent of mice treated by the diclofenac-loaded scaffolds showed recurrent tumor. In comparison, almost 90–100% of mice had recurrent tumor after surgery in the other groups. The survival rate of mice using diclofenac-released scaffold was 89%, while the survival rates of other groups were 10–25%. Immunohistochemical staining of recurrent tumors showed that the proliferation marker Ki-67 was reduced by nearly 10-fold in the tumor treated by diclofenac-released scaffolds (Figure 9C). In conclusion, electrospun nanofiber scaffolds loaded with anti-tumor drugs have been a class of promising alternatives for oral cancer treatment and recurrence prevention.

## Conclusions and Future Outlook

In the past few decades, the electrospinning technology has been widely used in the field of oral repair and regeneration due to its unique advantages. A wide range of raw materials can be used for fabricating electrospun fibers, such as synthetic polymer, natural biomaterials, and their combinations, to achieve complementary properties. According to the different types of oral diseases, the defect location needs to be repaired, and the degree of inflammation, electrospun nanofibrous scaffolds hold good feasibility and versatility to be designed and modified physically, chemically, and biologically, to achieve the performance we need. Moreover, a large number of long-term studies have proved that electrospun nanofibers can provide an appropriate microenvironment for cell proliferation and differentiation, and promote the regeneration of bone and periodontal tissues, etc.

Although electrospun nanofibers have attracted widespread attention in the field of oral disease therapy, in particular, periodontal medicine and implant bone repair, there are still many difficulties need to be overcame. For instance, current research mainly focused on the control of structures and properties of electrospun nanofiber scaffolds such as porosity, fiber diameter, mechanical property, and surface roughness, etc. It is still a challenge to achieve anti-inflammatory, bone formation, and periodontal tissue regeneration using one scaffold. Furthermore, in terms of the rational design of drug-loaded nanofibers scaffolds, how to maintain the bioactivity of the payloads as well as realize the mass production of these bioactive products are still a major concern for clinic use. It is suggested to screen and combine different drug delivery systems such as hydrogels and liposomes to improve the loading and sustained release of drugs. We also expect that electrospun fibers can be constructed to 3D scaffolds for the repair of bulk tissues such as alveolar bone and maxillofacial bone. In this regard, it will better to integrate electrospun fibers with techniques involving the fabrication of 3D scaffolds such as 3D bioprinting, gas foaming, and freeze drying. Through the use of tissue engineering strategies, we believe that electrospun fibers will fit a broad range of applications and market prospects in the field of oral disease therapy.
